# Imipenem-cilastatin-induced psychosis: a case report

**DOI:** 10.1186/s13256-016-0883-x

**Published:** 2016-04-27

**Authors:** Jacob Ninan, Gemy Maria George

**Affiliations:** 1 Department of Hospital Medicine, Mayo Clinic Health Systems Franciscan Healthcare, La Crosse, WI USA; 2 Internal Medicine Residency Program, John H Stroger Hospital of Cook County, Chicago, IL USA

**Keywords:** Psychosis, Adverse drug reaction, Imipenem-cilastatin, Antibiotics

## Abstract

**Background:**

Elderly patients, in particular, have been reported to develop psychiatric side effects from antibiotics. Clarithromycin, quinolones, sulfamethoxazole-trimethoprim, isoniazid, penicillin, and cephalosporins have been reported to cause psychosis. This case report bridges a void in the medical literature with regards to the psychiatric adverse effects of imipenem-cilastatin.

**Case presentation:**

A 64-year-old Hispanic man in septic shock due to urinary tract infection was initiated on imipenem-cilastatin and mechanically ventilated, following admission to hospital. His mentation was normal for 72 hours after extubation and discontinuation of sedatives and opioids, following which he was noted to be in acute psychosis. Our patient’s imipenem-cilastatin dose had been increased 24 hours prior to his violent visual and auditory hallucinations because his renal function had improved. The physical examination and laboratory tests did not reveal evidence of a new central nervous infection or endocrinopathy. His mentation improved after his antibiotic was switched to ceftriaxone, based on culture and sensitivity testing. Similar psychiatric symptoms developed 2 months later when he was treated with imipenem for a recurrent urinary tract infection. His symptoms again resolved with modification of his antibiotic regimen.

**Conclusions:**

Endocrine dysfunctions (thyroid, adrenal, and pituitary disorders) and toxic ingestions are medical disorders known to cause brief psychotic episodes. Fluoroquinolones, penicillins, and trimethoprim-sulfamethoxazole are common antibiotics associated with this rare adverse effect. Several pharmacokinetic hypotheses have been proposed for this adverse effect: (1) N-methyl-D-aspartate receptor hypofunctioning, (2) sequential blockade of folic acid production, (3) inhibition of prostaglandin E2 and proinflammatory cytokine production, (4) increased central dopamine turnover, and (5) accumulation of toxic levels of the drug. Pre-existing psychopathology, relevant comorbidities, slow acetylation status, and increased permeability of the blood–brain barrier have been suggested to make patients more prone to developing psychosis. According to the literature, this psychiatric manifestation resolves within 2 weeks of discontinuing the offending agent. There appears to be underreporting of the psychiatric manifestations of imipenem-cilastatin, contrary to post-marketing surveillance data. It is imperative that physicians recognize these psychiatric side effects of antibiotics, because they are a fundamental treatment option.

## Background

Acute brain dysfunction has been proven to cause negative clinical outcomes, including increased mortality, length of hospital stay, and cost of care. Sedatives, opioid medications, dihydropyridines, and antihistamines are common classes of medications known to cause delirium [1]. It has been reported that 12–39 % of cases of delirium are caused by medication alone [2–4]. Antibiotics have also been associated with the development of delirium. There is a very thin line separating delirium from psychosis. Elderly patients, in particular, have been reported to develop psychiatric side effects from antibiotics. The antibiotics well known to cause psychosis are penicillins, cephalosporins, trimethoprim-sulfamethoxazole, fluoroquinolones, and antitubercular medications. This case report bridges a void in medical literature with regards to the psychiatric adverse effects of imipenem-cilastatin.

## Case presentation

**Fig. 1:**
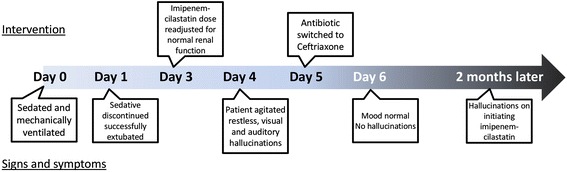
Timeline of development and resolution of symptoms with relevant events during the patient’s course of admission and readmission

A 64-year-old Hispanic man with traumatic paraplegia presented with a urinary tract infection (UTI). He had a neurogenic bladder and a history of recurrent UTIs but no prior history of psychiatric diseases. On examination, he was noted to have a temperature of 100.7 °F (38.17 °C), blood pressure of 93/56 mm Hg, a pulse of 147 beats per minute, and a respiratory rate of 28 breaths per minute. He had labored breathing, suprapubic tenderness, and frank pus in his urine when a urinary catheter was placed. He was emergently intubated and mechanically ventilated because his cardiorespiratory status deteriorated. At the time of admission, our patient’s white blood cell (WBC) count was 16,500/μL (neutrophils 75 %, lymphocyte 12.5 %, and bands 8.4 %). His biochemical parameters were within physiological limits except for his serum creatinine at 2.4 mg/dL (estimated glomerular filtration rate [eGFR], 25 mL/min/1.73 m^2^) and blood urea nitrogen (BUN) at 52 mg/dL. Two sets of blood culture and a urine culture grew *Klebsiella pneumoniae* (a final report including the culture antibiotic sensitivity was reported on day 4 of his hospitalization). A renally adjusted dose of imipenem-cilastatin was initiated because of his prior history of extended-spectrum beta-lactamase (ESBL) *Klebsiella* UTIs. Sedatives were discontinued, and our patient was successfully extubated after he demonstrated clinical improvement. Our patient was oriented to place, person, time, and situation after extubation; an assessment using the Confusion Assessment Method for the Intensive Care Unit (CAM-ICU) did not demonstrate any delirium. His family noted that he looked better and was conversing normally. The dose of imipenem-cilastatin was readjusted (increased) on his third day of hospitalization owing to the recovery of his renal function. On the fourth day of hospitalization, he was restless and agitated by paranoid thoughts (Fig. 1). He reported seeing and hearing people from his church planning to burn him at the stake.

The differential diagnoses entertained at this time were ICU delirium, encephalitis, endocrine dysfunction, structural neurological lesions (possibly stroke), or toxic ingestion. Our patient was oriented to place, person, and time with no new neurological deficits except for the visual and auditory hallucinations. Despite having an acute change in his baseline mental status (Richmond Agitation Sedation Score [RASS] 0 to 1+), he did not demonstrate any inattention (as demonstrated by picking ‘2’s) and, therefore, CAM-ICU was not diagnostic for ICU delirium. When our patient’s laboratory tests were repeated at the time of the psychotic symptoms, his WBC count was 9400/μL (neutrophils 74.2 %, lymphocyte 14.2 %). His biochemistry panel revealed serum creatinine of 0.9 mg/dL (eGFR, 65 mL/min/1.73 m^2^), BUN of 15 mg/dL, serum sodium of 138 mEq/L, serum potassium of 4.1 mEq/L, serum chloride of 102 mEq/L, and serum bicarbonate of 22 mEq/L. His 8 a.m. cortisol level was 18 μg/dL and his thyroid function was within physiological limits (TSH 1.04 mIU/L, free T4 7.4 μg/dL, and free T3 83 ng/dL). Repeat urine and blood cultures performed while he was experiencing psychotic symptoms were later reported to have tested negative for any infection. Further neurological imaging or invasive neurological procedures were not performed because he did not have any new focal neurological deficits.

Brief psychotic episode was diagnosed as per the fifth edition Diagnostic and Statistical Manual of Mental Disorders (DSM-5) criteria, and delirium was excluded because inattention could not be demonstrated. All reversible causes of psychosis were sought. The only change in the management of our patient was a change in the dose of imipenem-cilastatin; this was considered to be a possible etiology for psychosis. Objective evidence of a causal relationship between the drug and psychosis was assessed using the Naranjo Probability Scale. Our patient’s Naranjo adverse drug reaction (ADR) probability scale score was calculated to be +7; imipenem-cilastatin was the probable culprit [5]. Imipenem-cilastatin was discontinued, and he was switched to ceftriaxone according to susceptibilities from the blood and urine culture. His mood and behavior improved; all visual and auditory hallucinations resolved by his sixth day of hospitalization. He experienced similar psychiatric symptoms 2 months later when imipenem-cilastatin was initiated empirically for complicated UTI. On this readmission, the Naranjo ADR probability scale score was +9, definite, when he was rechallenged with imipenem-cilastatin. His hallucinations resolved within 48 hours of discontinuing imipenem-cilastatin, as in the first instance. Unfortunately, on both occasions, the levels of imipenem or cilastatin were not estimated owing to laboratory and logistic issues.

## Discussion

Prior to the third edition of the DSM, many terms existed to describe generalized brain dysfunction—acute confusional state, encephalopathy, acute brain failure, and ICU psychosis—causing terminological chaos. The American Psychiatric Association, since its third edition of the DSM, has worked diligently to update the definition to help clinicians identify delirium in patients of all ages. DSM-5 does not use the term “consciousness” but instead restrictively defines delirium in terms of attention, arousal, and cognition for the purpose of testability. Alterations in attention and awareness are core to the diagnosis of delirium. For example, despite an alteration in his level of consciousness (as evidenced by a change in RASS score), our patient did not demonstrate inattention, thereby excluding delirium. Brief psychotic disorder, as defined by DSM-5, is an episode of disturbance lasting at least a day but less than a month, characterized by one or more of the following: delusion, hallucination, disorganized speech, and grossly disorganized behavior. These symptoms should occur in the absence of a mood disorder or psychosis due to substance use/withdrawal or general medical condition. Our patient satisfied all the above criteria, with his symptoms occurring after his recovery from sepsis due to a UTI, when all his biochemical and microbiological tests were within normal limits.

Psychotic symptoms in patients being treated with antibiotics with non-central nervous system (CNS) infections have been reported in the past. The first documented case of neuropsychiatric symptoms following administration of antibiotics was noted with the intramuscular administration of penicillin in 1959 [6]. This disorder was characterized by severe psychomotor agitation with confusion, sensations of depersonalization and derealization, visual and auditory hallucinations, panic-like anxiety, and seizures. The term Hoigne’s syndrome was coined for an acute nonallergic reaction with the above-mentioned acute psychiatric symptoms to procaine penicillin.

The exact incidence of antibiotic-associated psychosis is not well known because of underreporting and an unknown volume of distribution of the medications. The majority of the associations between psychosis and antibiotic treatment in the medical literature are case reports. The common antibiotics associated with the development of psychosis are penicillins, cephalosporins, trimethoprim-sulfamethoxazole, fluoroquinolones, macrolides, and antituberculosis medications (Table 1).

**Table 1:** Common antibiotics causing psychosis

Class of Antibiotic	Name of Antibiotic
Penicillins	Amoxicillin
	Augmentin
Cephalosporin	Cefalexin
Aminoglycosides	Gentamicin
Tetracycline	Minocycline
Fluoroquinolones	Ciprofloxacin
	Ofloxacin
	Norfloxacin
	Temofloxacin
	Gatifloxacin
Sulfonamides	Trimethoprim-sulfamethoxazole
Metronidazole	
Macrolide	Clarithromycin
	Erythromycin
Antituberculosis medication	Isoniazid
Antivirals	Acyclovir
	Didanosine
	Zalcitabine
	Indinavir
Antifungal medication	Fluconazole

Infections are well known to be associated with acute psychosis. UTIs, in particular, have been noted to cause acute psychosis in geriatric patients. Manepalli *et al*. noted that 20 % of their geriatric patients with acute psychiatric illness had concomitant UTIs [7]. In a systematic review by Mostafa and Miller, a causal relationship was demonstrated between antibiotic treatment and psychosis in 60 % of the reported cases [8]. The common culprits associated with acute psychosis during the treatment of UTI were penicillin, trimethoprim-sulfamethoxazole, and fluoroquinolones. Eight of the 15 patients in the systematic review had a prior history of psychiatric disorders. In a paper published by the World Health Organization (WHO), seven of 82 patients reported to have manic episodes with antibiotics use were concurrently on mood-stabilizing agents [7]. In the systematic review, 80 % of the patients developed psychiatric symptoms within 1 week of initiation of the antibiotics [8]. The mean duration of psychotic symptoms was 5.1 days, and seven of the 15 patients required treatment with antipsychotics. Three of the 15 patients had a recurrence of symptoms when challenged with the same antibiotics.

The US Food and Drug Administration (FDA)-approved package insert for imipenem-cilastatin warns against CNS adverse effects such as seizures, confusional states, and myoclonic activity. The European Medicines Agency-approved package insert reports the frequency of hallucinations due to imipenem-cilastatin to be between 0.1 % and 1 %. The WHO Collaborating Center for International Drug monitoring, Uppsala Monitoring Centre, has 412 reports demonstrating a causal relationship between psychiatric disorders and imipenem-cilastatin sodium. The most common psychiatric disorder associated with imipenem-cilastatin was delirium (including confusion) with 208 reports, followed by disturbance in thinking and perception (including visual, auditory, or mixed hallucinations) with 105 reports [9]. However, a PubMed search {(“imipenem”[MeSH Terms] OR “imipenem”[All Fields]) AND ((((“mental disorders”[MeSH Terms] OR (“mental”[All Fields] AND “disorders”[All Fields]) OR “mental disorders”[All Fields] OR (“psychiatric”[All Fields] AND “disorder”[All Fields]) OR “psychiatric disorder”[All Fields]) OR (“hallucinations”[MeSH Terms] OR “hallucinations”[All Fields] OR “hallucination”[All Fields])) OR (“psychotic disorders”[MeSH Terms] OR (“psychotic”[All Fields] AND “disorders”[All Fields]) OR “psychotic disorders”[All Fields] OR “psychosis”[All Fields])) OR (“delirium”[MeSH Terms] OR “delirium”[All Fields]))} for articles related to imipenem-cilastatin causing psychiatric disorders failed to yield any results. This demonstrates an underreporting of the psychiatric adverse effects of imipenem-cilastatin in medical literature, contrary to post-marketing surveillance data. The early identification of these remediable psychiatric adverse effects could shorten the length of hospitalization, prevent avoidable mortality, and prevent eventual dementia.

There are various pharmacokinetic properties of the antibiotics proposed as hypotheses for these psychiatric adverse effects. GABA-A receptors are competitively inhibited by fluoroquinolones, causing an up-regulation of glutamatergic up-regulation; the resulting deficit in prefrontal-mediated executive function might induce psychosis [10]. Antibiotics cause the destruction of intestinal bacterial flora that produces D–alanine, which is essential for the normal functioning of the N-methyl-D-aspartate (NMDA) receptor [11]. Hypofunctioning NMDA receptors could cause psychosis, again by the loss of prefrontal control. The sequential blockade of folic acid production in the CNS has been postulated to cause the development of psychiatric symptoms in patients on sulfamethoxazole-trimethoprim. Psychosis associated with indomethacin has been attributed to the inhibition of prostaglandin E2 and proinflammatory cytokines. It has been postulated that macrolide antibiotics may also have similar anti-inflammatory properties, increasing central dopamine turnover, leading to psychosis [12].

## Conclusions

There is a very thin line between medication-induced delirium and medication-induced acute brief psychosis, but these conditions are reversible. Case reports record the resolution of psychiatric symptoms within 2 weeks of stopping the offending medication. Some patients may require neuroleptic medications. The treatment of this condition begins with identifying the culprit medication and suspension of the same. It is imperative that physicians recognize the psychiatric side effects of antibiotics because they are a fundamental treatment option.

## Consent

Written informed consent was obtained from the patient for publication of this case report and any accompanying images. A copy of the written consent is available for review by the Editor-in-Chief of this journal.

## Abbreviations

ADR: Adverse drug reaction
BUN: Blood urea nitrogen
CAM- ICU: Confusion Assessment Method for Intensive Care Unit
DSM-5: Diagnostic and Statistical Manual of Mental Disorders, 5th Edition
eGFR: Estimated glomerular filtration rate
ESBL: Extended spectrum beta-lactamase
NMDA: N-methyl-D-aspartate
RASS: Richmond Agitation Sedation Score
UTI: Urinary tract infection
WBC: White blood cell
